# Progress in State Medicine

**Published:** 1902-08-16

**Authors:** 


### PROGRESS IN STATE MEDICINE.

### VENTILATION.

BLYTH[1] thinks that there is little doubt that the standard of purity of the air which was introduced by the late Dr. de Chaumont, viz., that the air of living-rooms should contain under 6 parts per 10,000 of carbon-dioxide, is too high in the case of inhabited towns. A preferable standard for living-rooms would be that they should not contain more than 2 parts per 10,000 in excess of the outside air. An increase in temperature causes the air to expand, and hence to become lighter, the same mass now occupying a larger volume. Changes of barometric pressure are of much less importance in local ventilation, as their effect is over a large area, whereas differences of temperature constantly occur naturally, and can be easily produced artificially. These two natural forces are largely made use of in causing movements of air through tubes or pipes. These may simply be open to the external air, or the end may be fitted with some form of cowl or terminal to increase their efficiency. Numerous experiments have been made to test the efficacy of the various forms of cowls and terminals. It has been proved that the efficiency of a 3-inch pipe so fitted, compared with an open tube, was increased by 67 per cent., 13 per cent. gave results which were only slightly better, while 20 per cent. were inferior to the open tube. The results which are obtained by this method of ventilation are largely dependent upon the rate of movement of the external air across the open end of the shaft, as well as upon the difference of temperature between the external and internal air. It is on this account that ventilation by these means can be but little depended upon, as it is during high winds that the shaft will be most efficient; on dull, airless days, when ventilation is most required, it is least efficient. There are several simple forms of ventilating appliances for domestic use. The Hinckes Bird method, in which the lower window sash is partially raised, the lower opening being stopped by a board, has the disadvantage of causing unpleasant draughts near the window whenever there is much difference between the temperature of the external and internal air. The same disadvantage attends the use of Cooper's ventilators and other window appliances; when they act well, it is in weather in which the whole window had better be open. The various inlets connected with chimney flues, which are intended to extract air, are also usually unsatisfactory. Many patterns of ventilating grates act well, and in winter supply cheaply a large body of slightly warmed air. These grates are made with a cavity behind them which communicates directly with the external air, and delivers a current of warm air into the room by special openings which are situated most commonly just above the mantelpiece. The air in all rooms is in constant movement. In winter, for example, in rooms warmed by open fires, currents of warm air ascend to the ceiling from the more central parts of the apartment, while cool air streams constantly down the walls and window. In rooms lighted by gas three atmospheres exist: one near the ceiling, which is hot and impure; another near the floor, cool and pure, if derived from proper sources; while the intermediate layer is purer but less warm than the upper, and warmer and more impure than the lower layer. It is essential for efficient ventilation that the inlets and outlets for air should be placed in proper relative positions. The system of ventilation by fans has been applied by Lyon and Cadett to room ventilation. Large inlets and outlets are placed at opposite sides of the room. The air is delivered at a low and fairly equal velocity by one fan, and extracted by another. It is claimed that by this system draughtless ventilation can be effected in spaces of only 300 cubic feet per head; open fireplaces can be used, and the control of inlets and outlets maintained without closing either windows or doors. In middle-class houses ventilation is best secured for nine months of the year by open windows and doors. In cold weather the air in the hall and passages should be specially warmed. By this means unpleasant cold draughts are avoided, and on the return of warmer weather no condensation of moisture takes place on the walls. In an ordinary tenement house no mechanical means of ventilation is at present available. The cubic spaces sanctioned by statute in common lodging houses, in the cubicles of shelters, in tenement houses, in schools, factories, and workshops are impossible to ventilate by ordinary means so as to attain even a reasonable standard of purity. Attention[2] is drawn to the fact that a theatre or a large assembly room costs so much to build and keep in working order that it is comparatively easy to include in the outlay the expense necessary to secure more or less efficient ventilation, but that what is needed is some practical method of ventilating private houses. This problem will not be easily solved unless those concerned are willing to add to the ordinary cost of a private dwelling the cost of a scientific system of ventilation. Lyon calculates that each person should be supplied with 1,600 cubic feet of fresh air per hour, and this cannot be done regularly and in all weathers unless mechanical means are employed. A private room holding 12 persons would need 19,200 cubic feet of fresh air, and a motive power equal to moving one and one-eighth of a ton per hour. For ventilation to be perfect doors and windows must be shut and no air admitted save that which is pumped into the room, and then only is it possible to supply precisely the proper quantity at the desired temperature. For a small room the cost of electric power to drive the fans will not amount to more than 1d. per hour. The most difficult part is how to distribute this air in the rooms in such a manner as not to inconvenience the persons within. It is on these principles that Lyon ventilates his own dining room. Air is drawn in by a fan through a protected air inlet projecting through the roof. Before entering the room the air passes over trays containing mineral oil to arrest dust and soot, then over hot water coils. Thence it is driven by the fan into the room. To overcome the difficulty of equal distribution there is a conduit or shaft running along within the top of a false inner wall. The impetus given to the air by the fan is such that it will travel in a straight line to the end of the conduit, and not avail itself of the apertures on the road to enter the room. The greatest amount, therefore, escapes through the opening at the end of the conduit. To meet this difficulty, false inner walls have been built on two sides of the room facing each other, the one to serve for the inlet, and the other for the outlet of the air. The false wall, which can be made of wood, stands six inches in front of the real wall. Only a portion of this space, six inches deep, is required for ventilation, and the rest can be employed for real cupboards or for book-shelves. The part used for ventilating purposes has the appearance of closed cupboards. There are three of them on each of the two sides of the room. Plaster or panelling can be employed for these false cupboards, but behind this there must be a perforated shutter or wire gauze. The air propelled out of the horizontal conduit travels downwards to the bottom of the cupboard. Being checked it expands, and to prevent an undue amount escaping in this position, a baffling arrangement is placed behind the shutter. Strips of wood, in the shape of long acute triangles are fastened to the back of the shutter, with their broader part or base at the bottom of the shutter. They nearly touch each other, so that the apertures between them are smallest just where the pressure of the air is greatest, and become wider and wider as the upper part of the cupboard is reached. The principle applied within each cupboard is also applied with regard to the relationship of the cupboards the one to the other. Where the air enters at the beginning of the conduit with the greatest velocity, the cupboard is largest in order to offer the least resistance At the other end of the conduit, where the air is suddenly stopped, the nearest chamber is the smallest. For the air outlets there are similar air-chambers or cupboards on the opposite side of the room, only the baffling triangular pieces of wood are placed in the reverse way. The inlet apertures are largest at the top of the cupboards and the widest openings are at the bottom of the outlet cupboard. The hot air being propelled downwards meets the colder air, and these two currents create a rotary movement in the centre of the room. This prevents stratification. Communicating with the outlet cupboard there is also a horizontal conduit leading to a second fan which revolves in the reverse way to the other, thus acting as an exhaust. The air, though delivered in great volume and at considerable velocity, is so baffled and evenly distributed that its passage cannot be felt. It is objected that this system could not be applied to a room for much less than £150. Denison[3] states that a thorough knowledge of ventilation of a room necessitates an appreciation of the various activities of persons contained in the room; also of the extra equivalent space in a room needed to offset the rarefaction of the air at great altitudes; also the estimating of various diseased states—as when during fever additional poisons are thrown off into the air—and also the determining of the chemical and organic changes taking place in laboratories and workshops. The life of the air consists chiefly in its motion. This motion is always present till meddlesome man interferes. The study of the remarkable diffusability and penetrability of gases, even through glass windows and brick walls, not to mention wooden doors with the many cracks around them, gives some hope through what is called "insensible ventilation." There should be a legal limit for the minimum ventilation of all assembling places; places of habitation of both man and animals. He suggests for a living apartment a minimum space of 1,500 cubic feet per individual, and a minimum ventilation or change of air, of 2,000 feet per hour per person. If this much is not otherwise afforded in all new houses, then transoms over outside doors and windows, preferably opening inwards at their tops, should be required. A course for the study of ventilation should be made a prominent feature in all advanced schools and colleges. Until the establishment of a National Bureau, or Board of Health, Government ought to found and liberally maintain a commission on ventilation of houses, public halls, schools, factories and mines. The committee of the Manchester and Salford Sanitary Association,[4] have compiled some recommendations as to the ventilation of schools. Teachers are urged to make the best use of means provided for ventilation. Inattention to this important subject is to a large extent responsible for sore throats, anæmia, and mental dulness which occur among school children. In addition it favours the spread of infectious disease, and is extremely prejudicial to children who are predisposed to phthisis. Defective ventilation also leads to conditions of ill-health among teachers, especially to hoarseness, partial loss of voice, and undue mental weariness. Teachers should insist—(1) that foul air outlets are kept open at all times, the size of opening being regulated to minimise draughts; (2) that sufficient air inlets (which should be at a lower level than the outlets) are kept open to supply fresh air during teaching hours, and that the currents of air are of a "through" character; (3) that during playtime recess, dinner hour, before morning school, and after the close of the day's work all windows and doors be left open sufficiently long to "air" and refresh the schoolroom and classrooms; (4) that fire-places are not blocked up by fancy screens in summer, and that air inlet or air outlet gratings are left uncovered by maps, etc.;

(5) that each assistant teacher follow out and practise the same regulations; (6) that opportunity be taken to instruct the children in the value of ventilation both in school and for their own homes; (7) that the ventilation should be examined when much "school illness" is noted in the classes.

[1] Treatment, January 1902. [2] Lancet, March 1, 1902. [3] Jour. Balneology and Climatology, October 1901. [4] Brit. Med. Jour., March 8, 1902.

